# The Effects of Reprocessing and Fiber Treatments on the Properties of Polypropylene-Sugarcane Bagasse Biocomposites

**DOI:** 10.3390/polym12071440

**Published:** 2020-06-27

**Authors:** Juan P. Correa-Aguirre, Fernando Luna-Vera, Carolina Caicedo, Bairo Vera-Mondragón, Miguel A. Hidalgo-Salazar

**Affiliations:** 1 Research Group for Manufacturing Technologies GITEM, Universidad Autónoma de Occidente, Cali 760030, Colombia; jpcorrea@uao.edu.co; 2 Research Group for Development of Materials and Products GIDEMP, National Center for Technical Assistance to Industry (ASTIN-SENA), Cali 760003, Colombia; fernandolunavera@gmail.com (F.L.-V.); ccaicedo60@misena.edu.co (C.C.); bvera@sena.edu.co (B.V.-M.)

**Keywords:** biocomposites, recycling, rheological properties, DMA, injection molding

## Abstract

This study explores the reprocessing behavior of polypropylene-sugarcane bagasse biocomposites using neat and chemically treated bagasse fibers (20 wt.%). Biocomposites were reprocessed 5 times using the extrusion process followed by injection molding. The mechanical properties indicate that microfibers bagasse fibers addition and chemical treatments generate improvements in the mechanical properties, reaching the highest performance in the third cycle where the flexural modulus and flexural strength increase 57 and 12% in comparison with neat PP. differential scanning calorimetry (DSC) and TGA characterization show that bagasse fibers addition increases the crystallization temperature and thermal stability of the biocomposites 7 and 39 °C respectively, without disturbing the melting process of the PP phase for all extrusion cycles. The rheological test shows that viscosity values of PP and biocomposites decrease progressively with extrusion cycles; however, Cole–Cole plots, dynamic mechanical analysis (DMA), width at half maximum of tan delta peaks and SEM micrographs show that chemical treatments and reprocessing could improve fiber dispersion and fiber–matrix interaction. Based on these results, it can be concluded that recycling potential of polypropylene-sugarcane bagasse biocomposites is huge due to their mechanical, thermal and rheological performance resulting in advantages in terms of sustainability and life cycle impact of these materials.

## 1. Introduction

The reinforcement of polymers with natural fibers such as coir coconut, hemp, sisal, pineapple leaf fibers, sugarcane bagasse, fique and their combinations to create biocomposites has been studied in recent years [1,2,3,4,5,6,7]. The term biocomposites refers here to polymeric reinforced composites, where the reinforcing phase and/or the matrix are derived from materials of biological origin. In this sense, several studies have reported the formulation and characterization of biocomposites, which have a status of renewable and sustainable materials since they are composed of natural fibers embedded in non-degradable (i.e., polypropylene, polyethylene, polyamides, etc.) and biodegradable polymeric matrices (starch, polylactic acid, and polyhydroxialkanoates) [8,9].

These materials have the potential to replace traditional plastics in commercial applications such as car parts, toys, furniture, reusable cutlery, among others due to their low cost in comparison with traditional fibers and the enhancement of the polymeric matrices properties induced by natural fibers incorporation. These improvements include weight reduction, better specific properties, dimensional stability, biodegradability, recyclability, decrease in the embodied energy of the products, carbon emissions, and costs due to the polymeric substitution fraction that reduces the amount of plastic material needed to manufacture products [3,10,11,12,13,14].

Sugarcane is one of the most important crops for sugar production around the world. According to the Food and Agriculture Organization located in Rome, Italy (FAO), Colombia is the second-largest producer of sugarcane in South America, with an estimated 220,000 ha planted in 2019 [15], which produces approximately 6 million tons of bagasse by year [16]. This agro-industrial by-product is generated in sugar factories after the cane stem has been crushed and pressed. Sugarcane fiber is mainly composed of cellulose (37 wt.%), hemicellulose (21 wt.%), lignin (22 wt.%) and pectin (10 wt.%) [17]. The availability of this by-product, its low cost and the possibility of valorization are competitive advantages for the development of bagasse fibers based biocomposites at the regional level.

The combination of natural fibers with polymeric matrices generates a problem associated with the incompatibility between the polar and hygroscopic cellulose of the fibers and the non-polar and hydrophobic polymers. Additionally, other components of the natural fiber like hemicellulose, lignin, pectin and waxes generate a smooth surface that hinders the interlocking and the interfacial bonding between the matrix and the reinforcing phase [2,17].

For this reason, several researchers have performed surface treatments over the natural fibers to improve their compatibility with the polymeric matrix. These surface treatments could exhibit physical or chemical nature according to the mechanism applied to improve the interfacial bonding. The most used surface treatment methods included molecular interdiffusion, electrostatic bonding, mechanical interlocking and chemical modification through bleaching, acetylation, alkaline treatments and chemical bonding by coupling agents such as silanes or maleic anhydride [2,17,18].

Anggono et al. [19] studied the incorporation of bagasse (up to 30 wt.%) in a polypropylene (PP) matrix using injection molding processes. They perform alkali treatments on the fibers with calcium hydroxide (Ca(OH)_2_) and sodium hydroxide (NaOH) and evaluated the effect of those treatments on the mechanical properties of the biocomposites. The results showed that the tensile strength of the biocomposites increases proportionally with bagasse content and chemical modification of these fibers. Additionally, the biocomposites obtained from NaOH treated fibers present the highest mechanical performance results. Carvahlo et al. [20] studied the effect of bagasse content (up to 20 wt.%) and chemical modification (NaOH and acetylation) on the mechanical performance of recycled high-density polyethylene-(r-PE) biocomposites obtained by extrusion. Their results show that chemical modification increased the compatibility between r-PE and bagasse fibers and improves the mechanical properties of the biocomposites. Zainal et al. [21] studied the mechanical, thermal and morphological properties of biocomposites based on a recycled polypropylene-acrylonitrile rubber blend (PP-NBRr) and chemically modified bagasse fibers (up to 30 wt.%) with NaOH and silanes, prepared using melt blending techniques. Their results showed that chemical modification of the fibers enhances the thermal stability and tensile mechanical properties of the biocomposites. They also observed that among chemical treatments, silanization generate better results on the evaluated properties.

The reviewed literature showed that natural fiber-polyolefin based biocomposites could be processed using high-volume manufacturing processes such as extrusion and injection molding reproducibility and production capacity, advantages for the development of products using these materials. However, these manufacturing processes generate some scrap. In the case of injection molding, the overall process generates waste as gates, runners and sprues, which must be ground after the process. Thus, the recycling of wastes generated after products life cycle ending and during processing is an issue to study further and a lucrative option for the growing biocomposites industry that has not yet been fully explored.

Mechanical recycling of polyolefins like PP has been studied due to its ease of processing, property retention and availability [22]. Martín-Alfonso and Franco studied the recycling of PP using multiple extrusion cycles (up to 10 cycles) [23]. Their results showed that thermo-mechanical reprocessing generates a scission of the PP chains, which generates a progressive decrease in thermal stability, melting temperature, viscosity and viscoelastic properties with reprocessing cycles increase.

Regarding biocomposites, Uitterhaegen et al. [13] studied the mechanical behavior of biocomposites based on polyolefins (PP and Bio-PE) and coriander straw (up to 40 wt.%) ground and reprocessed 5 times using injection molding. The authors reported that mechanical properties did not decrease more than 10% through the reprocessing cycles, giving a high recycling potential to these polyolefin-based biocomposites. In another study, Chaitanya et al. [12] explored the recycling of biodegradable biocomposites based on polylactic acid (PLA) and alkaline treated sisal fibers (30 wt.%). The biocomposites were recycled using extrusion (8 cycles) and it was observed that mechanical properties gradually decreased until the third recycling cycle. Beyond these cycles, a significant reduction in properties was observed due to the decrease in PLA molecular weight and fibers attrition. From these results, the authors conclude that PLA-Sisal biocomposites can be recycled up to 3 times to make low to medium strength commercial products.

In the present research, PP-bagasse microfibers (untreated and chemically modified with NaOH and silanes) biocomposites were obtained through extrusion followed by injection molding processes. The mechanical, thermal, rheological and viscoelastic properties were evaluated and compared in order to understand the effect of chemical modification and reprocessing cycles (up to 5 times) on the microfibers dispersion on the biocomposites properties. We consider that the study of the performance of recycled biocomposites is an excellent contribution that supports the novelty of this article, bearing in mind that the interest in the design and manufacture of sustainable and highly recyclable products by injection molding with biocomposites based on natural fibers is increasing around the world.

## 2. Materials and Methods

### 2.1. Materials

PP reference 01H41 was sourced from Essentia (Cartagena, Colombia). Untreated sugarcane bagasse fibers were provided by Sucromiles S.A. (Cali, Colombia). In order to perform the chemical modification of these fibers, analytical-grade reagents hexadecyltrimethoxysilane and NaOH were obtained from Sigma-Aldrich (Milwaukee, WI, USA).

### 2.2. Methodology

#### 2.2.1. Preparation and Chemical Modification of Sugarcane Bagasse

The bagasse fibers were first washed with distilled water and dried at 60 °C for 48 h to remove soil and residues. Then, clean bagasse fibers were grounded with a lab mill and sieved through a 200 μm sieve. The bagasse fibers were separated into three groups: untreated bagasse, aqueous solution of 8% NaOH treated bagasse and aqueous solution of 8% NaOH following by silanized treated bagasse. The chemical surface treatments were performed according to the procedure described in detail in previous research work reported earlier by our group [6].

#### 2.2.2. Processing of Biocomposites

The reprocessing of the biocomposites was simulated using a continuous extrusions methodology. For this technique, the PP and the bagasse fibers were physically mixed in a bag using 20 wt.% of bagasse. This formulation was selected based on experimental results of our group and reviewed literature regarding the microinjection optimization of PP-Bagasse biocomposites [24]. This mixture was fed into the feed zone of a co-rotating twin-screw extruder HAAKE ™ PolyLab™ (Thermo Scientific-Unites States) with 16 mm diameter and 40 D total length, using a temperature gradient between 140 and 170 °C and a screw speed of 70 rpm. These processing parameters were selected from previously reported studies on polyolefin-based biocomposites [6,25]. Then, the extruded material was cooled in water and subsequently pelletized using a mechanical cutter that generated 5 mm long pellets. These pellets were dried in an air oven at 85 °C for 8 h after each extrusion cycle. The granules of neat PP and biocomposites were extruded 5 times, generating a total of 20 batches of granules that led to the development of 5 biocomposites and 10 reprocessed biocomposites. For this study, the properties of the materials corresponding to processing cycles 1, 3 and 5 were evaluated. The nomenclature of the prepared biocomposites is listed in Table 1.

**Figure 1:**
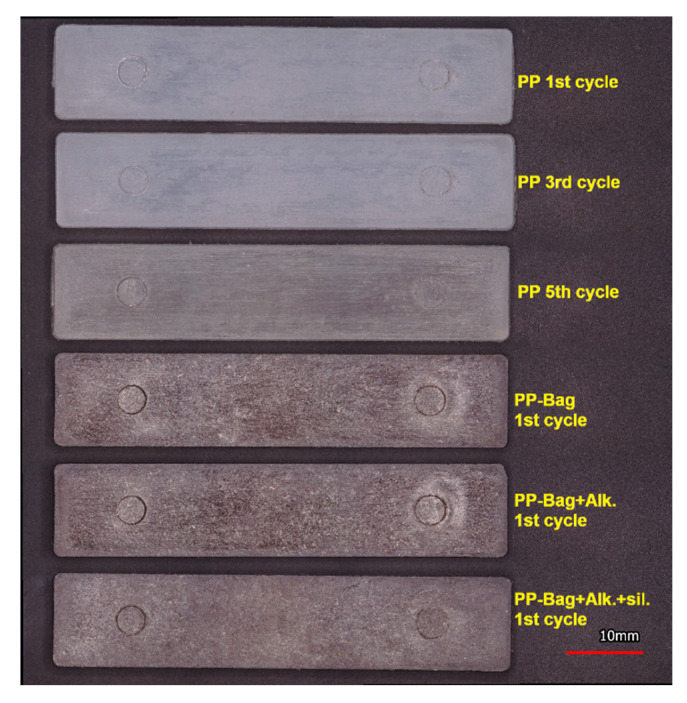
Injected specimens of PP and their bagasse fiber biocomposites.

Finally, a small quantity of the pellets of the different biocomposites was used for the development of injected specimens for flexural and impact tests using a BOY XS microinjection molding machine (BOY Machines Inc., United States) with a temperature gradient between 180 and 185 °C (from the feeding area to the nozzle), a filling pressure of 80 bars, a holding pressure of 60 bars and a mold clamping force of 30 kN. Figure 1 shows the injected PP specimens corresponding to 1st, 3rd and 5th processing cycles and the biocomposites of the 1st processing cycle. It is observed that after the 3rd processing cycle, the neat PP samples show a yellow shade that indicates thermal degradation of the matrix during reprocessing.

#### 2.2.3. Mechanical Properties

The mechanical properties in terms of flexural and impact performance of the materials were determined following the ASTM D790-17 and D256-10 standards, respectively. Three-point bending tests were performed using an INSTRON 3366 universal testing machine, while impact tests were performed using a 2.5 Joules impact tester. Flexural tests were carried out up to 5% deformation using specimens with a rectangular cross-section and 3.2 mm of thickness. The crosshead speed was 1.36 mm/min and the distance between the support span was 50 mm, while the impact tests were carried out on notched specimens (IZOD). The results were taken as the average of 5 samples and were subjected to an analysis of variance (ANOVA). Post hoc comparison was performed to determine the individual means, which are significantly different from a set of means of each reprocessing group using Tukey’s test at a 5% probability level.

#### 2.2.4. Thermal Measurements

The thermal stability of the materials was evaluated by thermogravimetric analysis (TGA), measuring the weight loss (%) as a function of temperature using a TA Q500 thermogravimetric analyzer (Texas Instruments, Dallas, TX, USA). These tests were carried out from 25 to 600 °C at a heating rate of 10 °C/min in a nitrogen atmosphere to determine the onset degradation temperature (T_o_) and the temperature at the maximum degradation rate (T_max_). In order to explore the effect of reprocessing cycles and chemical modification of the fibers on the thermal properties of the material, differential scanning calorimetry (DSC) tests were performed. These tests were carried out at a heating–cooling rate of 10 °C/min in a nitrogen atmosphere in several steps: First, the samples were subjected to heating cycle from 20 to 190 °C to erase the thermal history related to processing events, following by a cooling cycle from 190 to 0 °C to determine crystallization temperature (T_c_). Finally, a second heating cycle was performed from 0 to 200 °C to determine the melting temperature of the PP phase. Additionally, the degree of crystallinity (χ_c_) of each material was calculated from Equation (1) [26]:

(1) $$\chi_{c}=\left(\frac{\Delta H_{m}}{\Delta H_{m}^{0}\times W}\times 100\right)$$

where *W* represents the PP fraction by weight, Δ*H_m_* is the normalized melting enthalpy of PP of each sample, and $\Delta H_{m}^{0}$ (207 J/g) is the melting enthalpy of 100% crystalline PP [27].

#### 2.2.5. Rheological Measurements

The rheological behavior was determined by a rotational rheometer DHR-2 (Texas Instruments, Dallas, TX, USA) equipped with a cone-plate configuration with a diameter of 25 mm and an angle of 5.7°. For this geometry, the cone was truncated to avoid contact between the cone and the plate, and to prevent damage to either with a calibrated distance of 145 µm at the center of the cone. The rheological measurements were performed at 195 °C, the shear rate between 0.1 and 10 s^−1^ and a strain of 1%. Storage modulus (G′), loss modulus (G″) and complex viscosity (η*) were measured.

#### 2.2.6. Dynamic Mechanical Analysis (DMA)

The thermo-mechanical properties of the materials were evaluated using a dynamic mechanical analysis (DMA) RSA-G2 (Texas Instruments, Dallas, TX, USA) with a three-point bending clamp. The equipment was set up as follows: frequency of 1 Hz, 0.01% of strain, temperature range from −50 to 120 °C and a heating rate of 3 °C/min. Storage modulus (E′), loss modulus (E″) and tan δ (loss factor) were measured.

#### 2.2.7. Scanning Electronic Microscopy (SEM)

SEM of the different samples was carried out on the cryogenic fracture surfaces of non-tested injected specimens, operating at a voltage of 10 kV. The samples were previously sputter-coated with gold to increase their electric conductivity. Magnifications of 200× of the fracture surfaces were taken.

## 3. Results and Discussion

### 3.1. Mechanical Properties

The influence of bagasse fibers addition and reprocessing cycles on the PP flexural properties were evaluated. With ever increasing demand for high quality and reliable materials and products, flexural tests have become an important tool in both the manufacturing process and research fields to define the material ability to resist deformation under load [28]. Some recent studies have been carried out to study the effects of reprocessing on the flexural and tensile properties of PP reinforced with natural fibers [13,24]. Their results show some differences between flexural and tensile properties, the latter being lower than the former.

**Figure 2:**
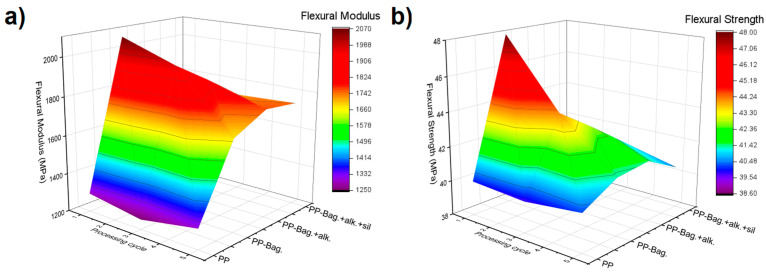
Three-dimensional colormap surface of flexural modulus (**a**) and flexural strength (**b**) of PP and PP-Bagasse biocomposites.

During a tensile test, the entire sample is under tensile stress and the rupture begins through the propagation of the largest defect within the specimen. On the other hand, during a flexural test, the maximum stress occurs at the upper and lower surfaces of the specimen where the shear stress is minimum. If the largest defect in the sample is not located in these sections, its influence on the failure mechanism and, therefore, on the flexural strength of the material will be minimal [13]. Therefore, the tensile strength values will be lower as compared to flexural strength values. Despite these differences, in these studies, it was observed that the results of both characterization techniques follow a similar trend with reprocessing cycles. Therefore, the flexural test has been validated as a valuable tool for the mechanical characterization of biocomposites. Figure 2 presents the three-dimensional colormap surface of the flexural modulus and flexural strength of the materials, which are summarized in the Table 2.

The 3D colormap surface indicates that successive reprocessing cycles did not affect the flexural behavior of the PP. However, bagasse fibers addition and chemical treatments performed on these fibers generate improvements in the flexural properties of the PP matrix. Additionally, it is observed that flexural behavior of biocomposites are dependent on reprocessing cycles of the materials, reaching maximum values around the third cycle. With subsequent reprocessing, a decrease in flexural properties was observed.

The first processing cycle shows the flexural modulus (FM) of biocomposites PP-Bag. and PP-Bag. + Alk, increased by 60% and 42% compared to neat PP. Additionally, flexural strength values (FS) increased by 20% and 8%, respectively. For biocomposite PP-Bag. +alk. +sil. FM value increased by 16%; however, no significant differences in the FS value were observed (*p* ≥ 0.05). Cerqueira et al. investigated the effect of untreated bagasse addition on the flexural properties of PP and found that FM and FS values increased by 32% and 35% respectively [29]. These improvements in flexural properties due to the addition of natural fibers have been observed in long [30] and short fibers [31,32]. However, it is essential to remark that our study demonstrated that this effect was also generated with the addition of microfibers.

For the third processing cycle, all FM and FS values of the biocomposites show significant differences compared to the FM value of the PP matrix. These increments were 57%, 48% and 38% for PP-Bag., PP-Bag. + Alk. and PP-Bag. +alk. +sil respectively. Additionally, FS values increased by 11%, 7% and 4% respectively. It is interesting to show that FM and FS values of the sample PP-Bag. +alk. +sil. increased by 15% and 7% in comparison with the first processing cycle values. This could be related to a better dispersion state of the silanized bagasse fibers within the PP matrix, the higher thermal stability of chemically modified fibers [6] and a better interaction fiber–matrix generated by the reprocessing cycles.

For the last reprocessing cycle, FM values of the biocomposites show significant differences in comparison with the PP matrix (*p* < 0.05); however, no significant differences were observed among the biocomposites. In the same way, the FS values of the samples were statistically equivalent. These results show that reprocessing could improve fiber dispersion and improve fiber–matrix interaction under compression stresses developed in the biocomposites during bending. However, these improvements seem to achieve a maximum point that in our study corresponded to the third cycle.

**Figure 3:**
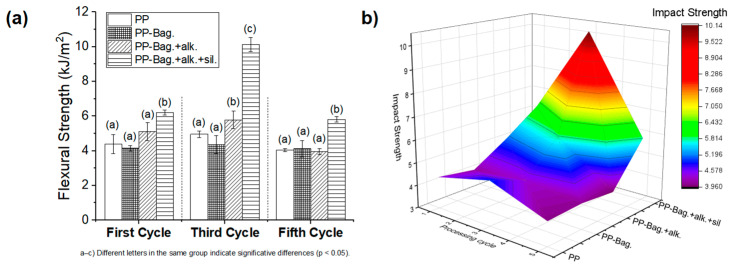
(**a**) Impact properties of PP and PP-Bagasse biocomposites and (**b**) 3D colormap surface of the impact properties.

Similar behavior was reported by Chaitanya et al., [12], who studied the recyclability of polylactic acid-sisal biocomposites. They found that reprocessing generates a severe reduction in mechanical and viscoelastic properties due to fiber and matrix degradation; therefore, they concluded that recycling of PLA/Sisal biocomposites beyond third reprocessing cycle is not recommended. Figure 3 shows the effect of the addition of bagasse fibers and the reprocessing cycles on the impact strength values.

For the first processing cycle, no significant differences were observed in the impact strength values of the PP matrix and the biocomposites PP-Bag. and PP-Bag. + Alk. However, for the PP-Bag. + Alk. +sil. biocomposite the impact strength increased by around 40% compared to neat PP. This result shows that bagasse fibers treated by silanes agents had improved the capacity of the polymeric matrix to absorb energy. From the revised literature, it can be observed that several factors governed the impact behavior of natural fiber-reinforced biocomposites, for example, chemical treatment applied on the natural fibers, type of natural fiber, interfacial bonding, the composition of the biocomposite and the toughness of the polymeric matrix. In the case of silanes treatments, it was found that this process may have different effects on the impact properties of biocomposite PE-Hemp and PE-Sisal [17].

Biocomposites reprocessing causes interesting changes in the impact properties studied. According to the 3D colormap surface (Figure 3b), the third reprocessing cycle generates a significant increase in the impact values of PP-Bag. + Alk. and PP-Bag. + Alk. +sil. biocomposites. These increases were between 17 and 103% in comparison with reprocessed PP. For the fifth processing cycle, the impact values have similar behavior to that observed in the first cycle. The impact value of biocomposite PP-Bag. + Alk. +sil. increased by 43% compared to the PP. These results are evidence that reprocessing improves the dispersion state of the silanized fibers, fiber–matrix interaction and promotes PP energy absorption. However, as observed in the flexural test these improvements reach their highest point around the third reprocessing cycle.

### 3.2. Thermal Properties

**Figure 4:**
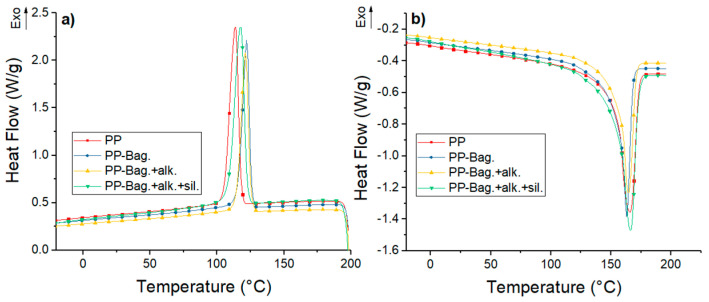
(**a**) Cooling and (**b**) second heating DSC curves for first processing cycle PP and PP-Bagasse biocomposites.

**Figure 5:**
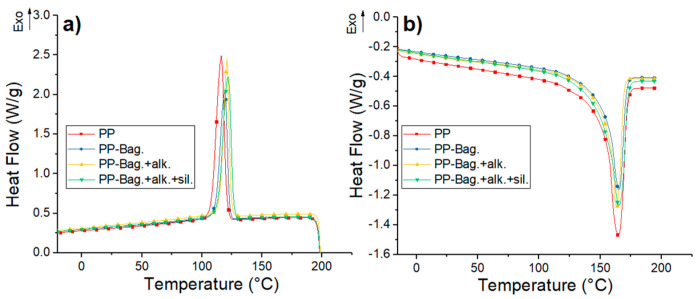
(**a**) Cooling and (**b**) second heating DSC curves for third processing cycle PP and PP-Bagasse biocomposites.

**Figure 6:**
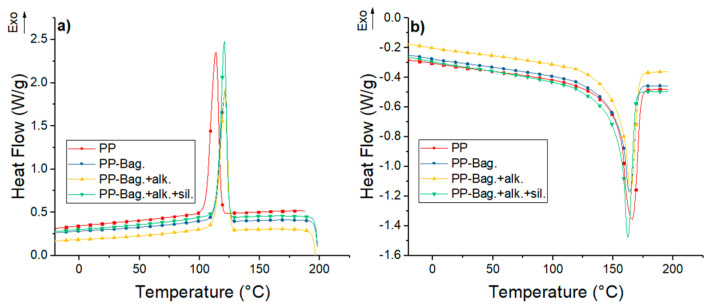
(**a**) Cooling and (**b**) second heating DSC curves for fifth processing cycle PP and PP-Bagasse biocomposites.

DSC curves for neat PP and their biocomposites with bagasse fibers at the 1st, 3rd and 5th processing cycles are shown in Figure 4, Figure 5 and Figure 6. The numerical values of the thermal events of the samples are shown in Table 3. These thermograms do not show any indication of bagasse fibers because the peaks attributed to the different reactions or mechanisms involved in pyrolyzing of the bagasse appears at temperatures higher than those selected for our DSC tests (above 290 °C) [33,34]. However, the fibers effect on the crystallization and melting behavior of the PP phase are discussed in this work.

Cooling thermograms of PP show exothermic peaks located between 114 and 116 °C. These peaks corresponded to the crystallization during the cooling of the PP chains. These crystallization peaks are also observed in PP-Bagasse biocomposites, however, these peaks are located at temperatures between 3 and 7 °C higher compared to the PP in the different extrusion cycles. This shows that bagasse fibers could act as nucleation points that allow the crystallization of PP chains at higher temperatures.

The second heating runs of PP and PP-Bagasse biocomposites present endothermic peaks between 163 and 166 °C related to the melting of the PP matrix. This indicates that bagasse fibers addition did not interfere with the melting process of the PP matrix. In this study, the maximum processing temperature was 185 °C, which is higher than PP melting temperature. This was done with the aim to ensure completely melting of PP crystals and improving the processing of the material without causing degradation to the bagasse fibers. In this aspect, some authors reported that biocomposites processing must be performed below 200 °C to avoid natural fibers degradation [2,17,35].

Additionally, melting enthalpy (ΔH_m_) and crystallinity degree (χ_c_) values of the biocomposites changed with the number of extrusion cycles and with the bagasse fiber type. For all extrusion cycles, a decrease in the ΔH_m_ values of PP-Bag and PP-Bag.-alk biocomposites were observed. However, the χ_c_ of the PP matrix remained similar when the ΔH_m_ was corrected, considering the weight fraction of bagasse (Equation (1)). This behavior was also observed in other natural fiber-polyolefin biocomposites [5,36]. On the other hand, for biocomposites with silanized bagasse fibers (extrusion cycles 1 and 3), an increase in the χ_c_ values of around 15% was observed in comparison with the χ_c_ of the PP matrix. This χ_c_ increase was slightly for cycle 5; however, it is concluded that the silanized process improved the nucleating effect of the bagasse fibers in the PP. Therefore, the mechanical strength improvement observed in the PP-Bagasse biocomposites could be related to the reinforcement effect of bagasse fibers in the PP and the crystallinity changes of the thermoplastic matrix. Similar results were reported by Zainal et al. [21] on polypropylene-acrylonitrile butadiene rubber-modified bagasse biocomposites. They reported that the chemical treatment of bagasse fibers using silanes increases the nucleation density and the crystallinity degree (%) of the polymeric matrix.

**Figure 7:**
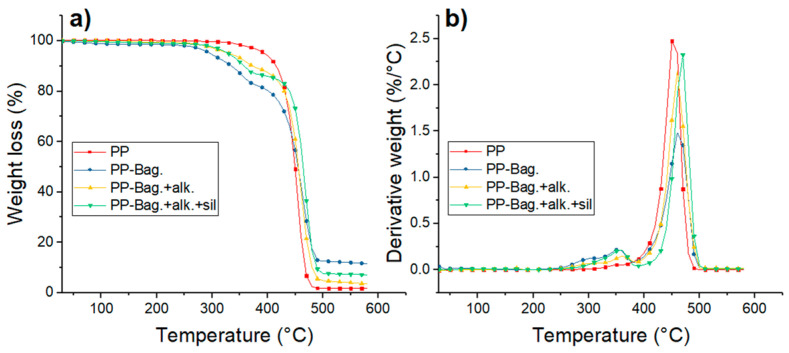
(**a**) TG and (**b**) DTG curves for first processing cycle PP and PP-Bagasse biocomposites.

**Figure 8:**
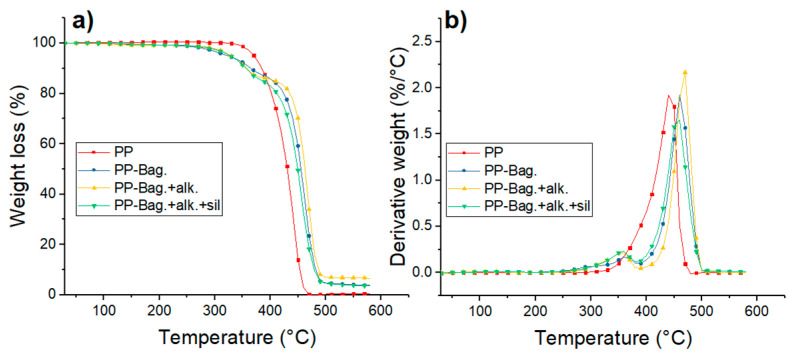
(**a**) TG and (**b**) DTG curves for third processing cycle PP and PP-Bagasse biocomposites.

**Figure 9:**
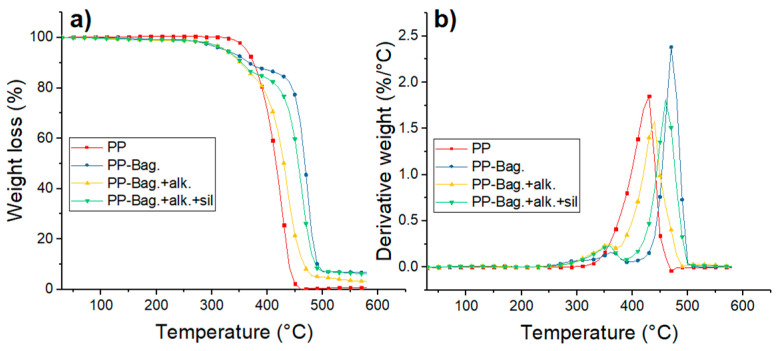
(**a**) TG and (**b**) DTG curves for fifth processing cycle PP and PP-Bagasse biocomposites.

Thermogravimetry (TG) and Derivative Thermogravimetry (DTG) thermograms of PP and PP-Bagasse biocomposites at the 1st, 3rd and 5th processing cycles are shown in Figure 7, Figure 8 and Figure 9. Additionally, the main thermal parameters obtained from these curves are summarized in Table 4.

The thermal degradation of PP matrices occurs in a single step process. For the first cycle, a T_o_ of 408 °C and a T_max_ of 455 °C were observed. For reprocessing cycles 3 and 5, To values decreased by 35 and 37 °C while T_max_ decreased by 10 and 24 °C as compared with PP at the first extrusion cycle. This lowering in PP thermal stability with melt reprocessing has been already observed in other studies and could be related to the chain scission mechanism of PP during multiple extrusions [23,37]. Da costa et al. cited that scission of the PP chains during reprocessing generates small and defective molecules, a broader distribution of molecular weights and reduction in the onset degradation temperature of the polymer [38].

For biocomposites, degradation occurs in a two-step process. The first step is related to the decomposition of the bagasse fibers within the biocomposite, while the second step corresponds to the thermal degradation of the PP matrix [6]. The first degradation step show that thermal stability of the chemically treated bagasse fibers is higher than the exhibited by untreated fibers. According to a previous research work published recently by our group the performed chemical treatments could help to extract low thermal stability components of the bagasse fibers like hemicellulose, lignin, pectin and waxes [6]. With only cellulose, the bagasse fibers gain some thermal stability. Additionally, the silanes presence increases thermal stability of the bagasse fibers within the biocomposite, mostly due to the formation of refractory siloxane networks between the fibers and PP after silanization as indicated by literature [39].

The second degradation stage shows that bagasse addition increases the thermal stability of the PP phase. For the first reprocessing cycle, To increased between 15 and 39 °C. Additionally, Tmax increased between 13 and 6 °C in comparison to neat PP as shown in Table 4. This increment in the thermal stability of the biocomposites has been observed in several studies and could be related to the increase of the crystallinity with bagasse addition observed by DSC [40,41]. This behavior is more evident during reprocessing cycles 3 and 5 due to the observed decrease of the thermal stability of neat PP during melt reprocessing. This result shows that bagasse addition improves the thermal stability of the polymer matrix when reprocessing cycles, such as mechanical recycling, are carried out.

After 500 °C, the residue of the samples remains. These residues were composed mainly of ashes and had a weight of 12% for PP-Bag., 8% for PP-Bag. +alk. +sil. and 4% for PP-Bag. +alk. This difference could be related to lignin present on the untreated bagasse, which generates a large number of solid residues after the pyrolysis of the fiber [33].

### 3.3. Rheological Properties

**Figure 10:**
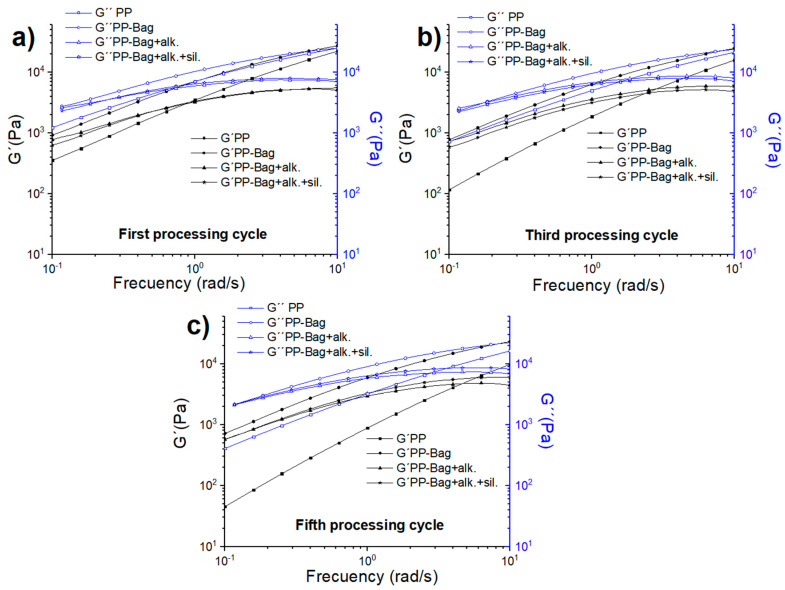
Storage and loss modulus as a frequency function of PP and PP-Bagasse biocomposites: (**a**) First, (**b**) Third and (**c**) Fifth processing cycles.

The influence of bagasse fibers addition and reprocessing cycles on the PP storage modulus (G′) and loss modulus (G″) modulus vs. a frequency is presented in Figure 10. According to Osswald and Rudolph, G′ is a measure for the stored energy and is related to the rigidity and relative entanglement of polymeric chains. On the other hand, G″ is a measure for the lost energy dissipated, for example, as heat or used on the relative movement among polymeric chains [42].

The results show that G″ is higher than G′ for neat PP and all biocomposites, indicating that these materials present a dominant liquid viscoelastic behavior in the studied frequency range. This behavior has previously been observed in another polyolefin-natural fiber biocomposites with fiber percentages up to 20% by weight [43,44]. Additionally, it is observed that G′ and G″ values of neat PP and biocomposites decrease with successive reprocessing cycles. This decrease has been previously observed and could be related to changes in the length and entanglements of PP polymeric chains caused by multiple extrusion processes [23]. Therefore, the elastic behavior of the biocomposites would be lower with successive reprocessing cycles.

Besides this, a decrease in G′ and G″ values are observed in biocomposites with chemically modified bagasse fibers. These chemical treatments modify the surface of the bagasse fibers; in general terms, these modifications reduce the particle agglomeration, improving the slip or flow between them inside the biocomposite. This is reflected in lower G′ and G″ values. It is interesting to note that the biocomposites with silanized bagasse fibers present the lowest G′ and G″ values for all reprocessing cycles. This could be due to short silane chains that could act as a lubricant at the PP-bagasse fibers interface and could reduce the internal stress generated by fibers agglomeration.

**Figure 11:**
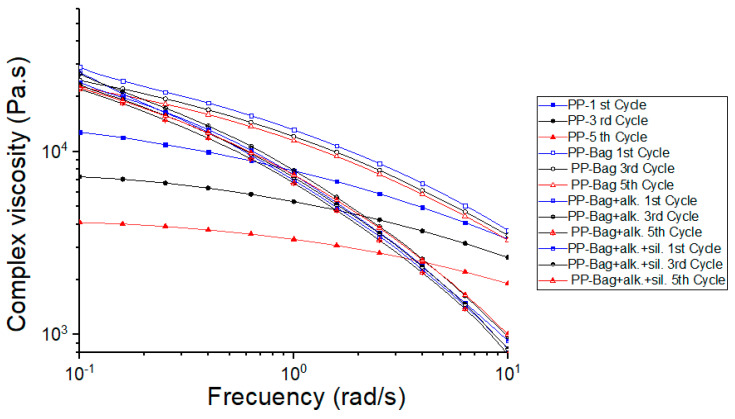
Complex viscosity as a frequency function of PP and PP-Bagasse biocomposites.

Figure 11 provides the complex viscosity vs. frequency of the neat PP and biocomposites. All materials show shear-thinning behavior, as had been previously observed in PP and PP-natural fiber biocomposites [43]. This behavior is related to the viscoelastic nature of the polymeric matrix and the interaction with the bagasse fibers. At the frequencies studied, the polymeric chains do not have enough recovery time due to the contact between the fibers leading to the non-Newtonian rheological characteristics observed.

The results also show that PP-Bag. biocomposites present the higher viscosity values in the entire frequency range studied. For PP-Bag., the relative movement and disentanglement of bagasse fibers are impeded due to agglomeration of fibers, which hinders polymer chains flow and increasing the viscosity values. For biocomposites obtained from chemically modified bagasse fibers, the viscosity values are lower in comparison to PP-Bag. biocomposites. As mentioned above, the untreated fibers can agglomerate due to adhesive forces between fibers. The performed alkaline treatment aimed to extract the lignin, which is a hydrophobic layer that covers the bagasse fibers. This treatment exposed the cellulose of the bagasse and improved their dispersion within the polymeric matrix, thus decreased the particle–particle interactions, allowing the polymer chains to flow and decreased the viscosity. Furthermore, the silanes treatment produced a functionalized surface with covalent Si-O bonds, which hindered the agglomeration of the fibers and acted as a lubricant, which improved the fibers flowing within the polymeric matrix, causing a decrease in the viscosity values and eased the processability of the biocomposites by conventional plastic transformation processes such as extrusion or injection molding.

Regarding the reprocessing cycles, it is observed that the viscosity values of PP and biocomposites decreased progressively with extrusion cycles. This decrease of the viscosity values was also reported on several PP reprocessing studies and can be related to a decrease of the PP matrix molecular weight due to polymeric chain scission during the multiple extrusion reprocessing steps [23,37,38].

**Figure 12:**
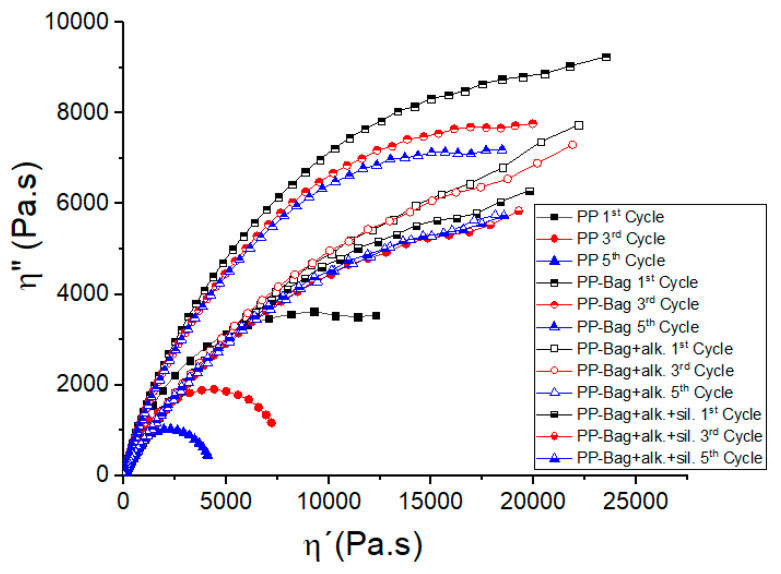
Cole–Cole plots of PP and PP-Bagasse biocomposites.

With the aim of further investigate the effect of reprocessing and bagasse fibers addition on the rheological and structural behavior of the materials, Cole–Cole diagrams were used (Figure 12). In this diagram, the imaginary viscosity component (η″) is represented as a function of the real component of the viscosity (η′). The graph should be like a semicircle if the system describes a single relaxation. In heterogeneous melts containing agglomerated fibers, the semicircle shape of the Cole–Cole graph will be modified, the elastic component of the viscosity, and the relaxation time increases [42].

The Cole–Cole plots of neat PP revealed a semicircle related to single relaxation time. On the other hand, untreated bagasse fibers addition generated an increase in the elastic behavior and relaxation time of the structure, visualized in viscosity components values increments. This behavior indicates the presence of agglomerated fibers and decreased progressively with extrusion cycles. Finally, it is observed that chemical modification of bagasse fibers clearly generated a decrease in the elastic component of the viscosity (n″) and shorter relaxation times of the materials. These results could indicate that continuous extrusion processes and chemical modification generate a better dispersion of bagasse fibers within the polymeric matrix.

### 3.4. Dynamic Mechanical Analysis

According to Saba et al., [45], the storage modulus (E′) is related to the ability of a material to store energy during a dynamic test and determine the stiffness of the sample. Additionally, E′ is essential for the evaluation of the mechanical properties from the molecular basis because it is sensitive to structural changes within the polymeric matrix such as molecular weight and the interfacial bond between the fibers and the matrix.

**Figure 13:**
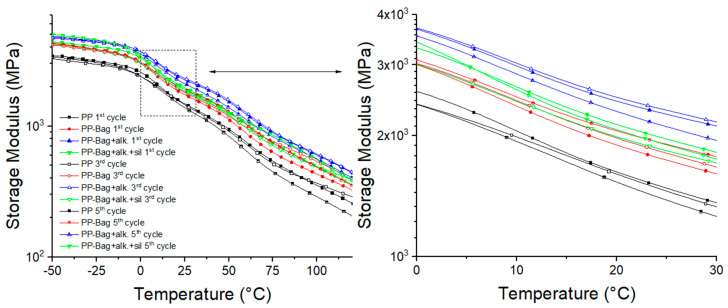
Temperature dependence of the storage modulus of PP and their biocomposites.

Figure 13 shows the E′ as a function of temperature for the first processing cycle. It can be observed that E′ values of PP increase after the bagasse fibers incorporation. At room temperature, the E′ value of neat PP (1481 MPa) increased to 18% with the addition of untreated bagasse fibers. This stiffness increase could be attributed to a decrease in the PP chains mobility generated by the rigid bagasse fibers and indicates that PP-Bag biocomposite had a higher capacity to store energy in comparison with neat PP. This behavior was even more significant after the incorporation of chemically modified bagasse. E′ value increased by 32 and 52% for PP-Bag. +alk. +sil. and PP-Bag. +alk. in comparison with neat PP, respectively. These results show that chemical modifications induced a better adhesion on the interface between bagasse fibers and PP matrix and increased the material capacity to absorb energy. This result was in good agreement with the data obtained from the impact test (Section 3.1).

Regarding reprocessing cycles, it is observed that E′ values of biocomposites increased progressively with extrusion cycles in comparison with neat PP. For the third reprocessing cycle, E′ values increased up to 27 and 60% for PP-Bag and PP-Bag. +alk., whereas, for the fifth reprocessing cycle, this increment lay between 35 and 57% (again for PP-Bag and PP-Bag. +alk.). This processing event has been already observed during PLA-Sisal biocomposites recycling and indicates that thermo-mechanical reprocessing generate an improvement in the interfacial bonding between the bagasse fibers and the PP matrix [12].

**Figure 14:**
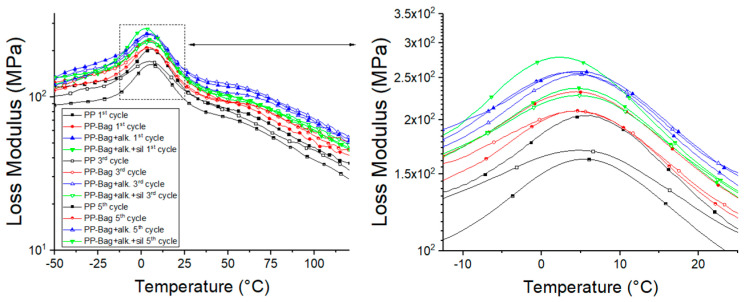
Temperature dependence of loss modulus of PP and their biocomposites.

Similar to the observed behavior in E′ graphs, the E″ peaks of PP-bag biocomposites were higher as compared to neat PP (Figure 14). These increases indicate a reduction in the mobility of PP chains due to bagasse fibers. These increases of E″ peaks were higher upon chemically modified bagasse incorporation for all reprocessing cycles and could be related to the enhanced adhesion at the interphase given by the chemical treatments, which suppressed the molecular mobility of the polymeric matrix. This trend has been observed for several polymer-chemical treated fibers biocomposites [6,46,47].

**Figure 15:**
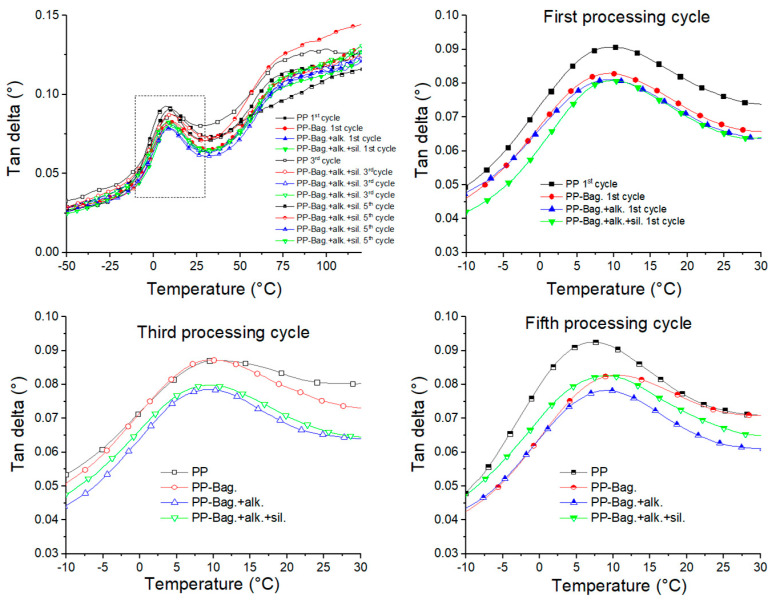
Temperature dependence of tan delta of neat PP and their biocomposites.

Tan δ is defined as the ratio between the loss and storage modulus (Tan δ = E″/E′) and is related to the damping properties of the polymeric matrix [45]. The variation of tan δ with temperature is represented in Figure 15. According to this graph, PP shows two main relaxation peaks in the evaluated temperature range, a β relaxation, located around 7–9 °C, which corresponds to the glass transition (Tg) and an α relaxation between 60 and 75 °C [6].

Table 5 shows that after untreated bagasse fibers addition into neat PP, Tan δ peaks height decreased for all reprocessing cycles. This is because bagasse fibers hinder the mobility of the polymer chains; also, these fibers support higher stresses fields, which generate less deformation of them at the interface, which causes more energy dissipation. Additionally, it is observed that bagasse fibers addition did not generate significant changes in PP relaxations values (Table 5). For biocomposites obtained from chemically modified bagasse fibers, the Tan δ peaks exhibited even lower magnitude when compared with untreated bagasse biocomposites for all reprocessing cycles. These results, albeit small, show that biocomposites with improved interfacial bonding between natural fibers and PP matrix, given by the chemical modification, will tend to dissipate less energy, showing the lower magnitude of the Tan δ peak in comparison with biocomposites with a weakly bonded interface and can be decisive in product applications that require better mechanical behavior under flexural loads.

To further understand the mobility of the chain segments and the state of dispersion, the full width at half maximum (FWHM) of tan δ peaks was evaluated. Some studies on biocomposites viscoelastic properties have shown that FWHM is a measurement of tan δ curve broadness and could be useful to evaluate the reduction of the molecular mobility during a relaxation like the glass transition [48,49]. According to Manikandan et al., a higher FWHM value implies more interaction and contact between the phases of the composite, which can be associated with low dispersion and a heterogeneous amorphous phase [49]. Among biocomposites, those based on untreated bagasse fibers present the higher FWHM values for all reprocessing cycles. This could be attributed to the suppressed effect of the poor dispersed fibers on the PP matrix molecular mobility. It is also observed that FWHM values decreased for all biocomposites with reprocessing, and it was found to be the lowest for chemically treated fiber-based biocomposites. This result shows that the successive reprocessing improved the homogeneity of the matrix and confirmed that chemical treatments improved the adhesion at the interface, which suppressed the molecular movement of the polymer matrix. This means that biocomposites obtained from chemically modified fibers acted more elastic and confirmed that under a load, these biocomposites had more potential to store energy instead of dissipating it.

### 3.5. Morphology

**Figure 16:**
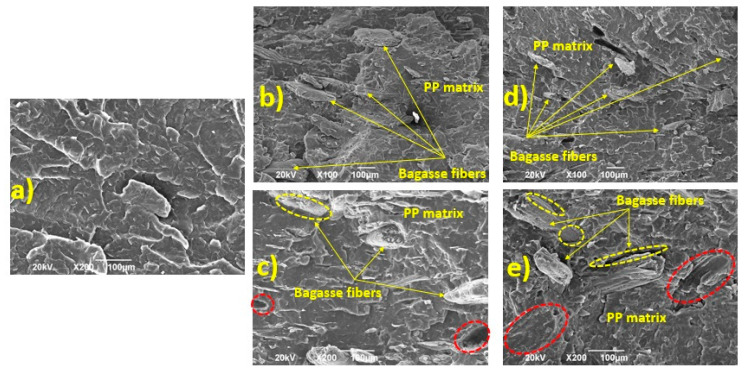
SEM micrograph of the fracture surface of (**a**) neat PP, (**b**,**c**) PP-Bag. 1st cycle and (**d**,**e**) PP-Bag. 5th cycle.

Figure 16 shows the cryogenic fracture surface of neat PP (Figure 16a) and PP-bag. Biocomposites at the first (Figure 16b,c) and fifth reprocessing cycles (Figure 16d,e). Neat PP presents an irregular surface fracture, which is caused by the inherent semi-crystalline structure of this polymer whereas untreated bagasse incorporation generates a rough fracture surface.

Regarding the first cycle, the lower magnification allows one to observe bundles of bagasse fibers within the PP matrix with an average length of 213 ± 24 micrometers (Figure 16b). On the other hand, higher magnification (Figure 16c) show some voids resulting from the fibers pulled out from the matrix (as shown in red circles) and well-delineated gaps at the interface between the bagasse fibers and the PP matrix (yellow circles). These gaps are evidence of the low interfacial adhesion between the PP and the untreated bagasse fibers. This microstructural behavior at the interface was observed in different polymer matrix biocomposites and was related to the low chemical affinity between the inherent hydrophobic polyolefin matrix and the hydrophilic natural fiber.

After five reprocessing cycles, a better distribution of the fibers and a decrease in their length to 165 ± 17 micrometers was observed (Figure 16c). This result shows that during the five extrusion and injection cycles the fibers get crushed to some extent and their length was reduced by 20% and could be related to the decrease observed in the mechanical performance observed in PP-bag. biocomposites considering all reprocessing cycles (Table 2).

Several studies have shown that reprocessing of biocomposites generates a decrease of 60% in the fibers length. The fibers length decreasing is higher than that observed in our study, however the initial size of the fibers used must be considered. In these studies, the initial size of the fibers was 1–0.8 mm, while in our study, the initial average size was around 0.2 mm. However, the authors report that biocomposites retain its mechanical performance throughout cycles better than fiberglass reinforced composites due to the inherent flexibility of the natural fibers and the ability to resist external mechanical forces.

Reprocessing cycles will inevitably impact the mechanical properties of biocomposites; however, our study shows that PP-Bag. biocomposites’ mechanical performance was maintained up to three reprocessing cycles without the addition of virgin material. Based on this result, it could be concluded that recycling potential of biocomposites was huge due to their mechanical performance retention resulting in advantages in terms of sustainability and life cycle impact of these materials.

**Figure 17:**
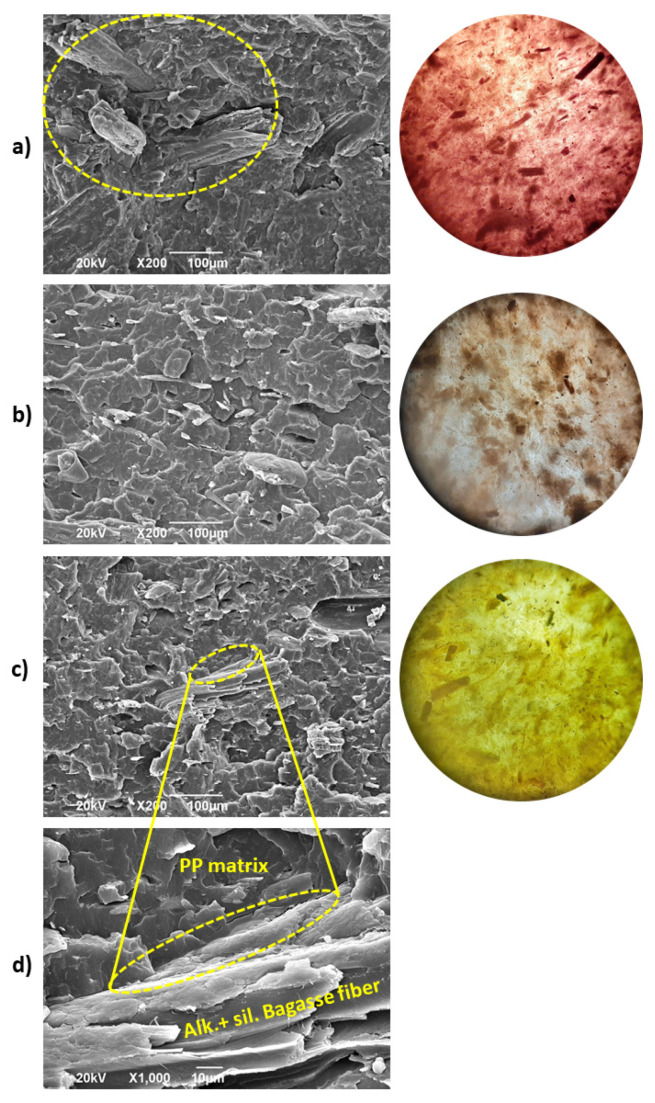
SEM (**left**) and optical micrographs (**right**) for 5th processing cycle: (**a**) PP.Bag., (**b**) PP-Bag + Alk. and PP-Bag + Alk. +sil. (**c**,**d**).

Figure 17 shows the cryogenic fracture surface corresponding to the biocomposites of the fifth reprocessing cycle along with the optical micrograph of the surface of each sample at the same lighting level. For Figure 17b,c, chemical treatments reduced the gaps between the bagasse fibers and the PP matrix and improved the interface of the biocomposites. Additionally, the optical micrographs of chemically modified fibers based biocomposites show a decrease of the dark areas related to particles agglomeration on the surface of the sample. This behavior is observed in better detail in the sample with silanized bagasse and could be due to the lubricant effect given by silanes, which improved the dispersion of the fibers. This result was also consistent with the data obtained by rheology and DMA measurements and confirmed that chemical treatments generated a bonding effect at the PP and natural fibers interface and exposed the cellulose of the bagasse improving their dispersion within the polymeric matrix.

The scanning electronic microscopy gives valuable information about biocomposites morphological characterization. Several authors have made conclusions studying the fiber content (composition), the chemical treatment of natural fibers, and biocomposites reprocessing using SEM [12,20,21,44]. This technique, together with the performed rheological and dynamic mechanical characterization, could provide valuable evidence of the dispersion state of the fibers within the biocomposites. In our case, the Cole–Cole plots and FWHM of tan δ peaks gave evidence for concluding that multiple extrusion cycles could decrease the particle agglomeration and generated a better dispersion of the fibers within the matrix.

## 4. Conclusions

In this research, PP-bagasse biocomposites were prepared by incorporating 20% by weight of bagasse fibers treated by alkaline treatment with NaOH and silanization after the alkaline treatment.

These biocomposites were reprocessed 5 times using the extrusion process followed by injection molding after each reprocessing cycle in order to evaluate the effects of reprocessing and chemical treatments on the morphology, mechanical, thermal, as well as viscoelastic properties of these materials. Doing so, the following conclusions could be obtained from the present research:

- The mechanical properties indicate that reprocessing and chemical treatments performed to bagasse microfibers could improve fiber dispersion and fiber–matrix interaction under compression stresses developed in the biocomposites during bending, and promoted PP energy absorption. These mechanical improvements achieved a maximum point that, in our study, corresponded to the third cycle.
- Thermal characterization revealed that bagasse fibers addition increased the crystallization temperature and the thermal stability of the PP phase for all extrusion cycles without disturbing the melting process of the PP matrix. Additionally, silanized fibers based biocomposites presented the highest thermal stability for all processing cycles.
- The rheological test shows that the viscosity values of PP and biocomposites decreased progressively with extrusion cycles. Additionally, biocomposites obtained from chemically modified bagasse particles presented lower viscosity values in comparison with neat bagasse based biocomposites. However, Cole–Cole plots indicates that continuous extrusion processes and chemical modification generated a better dispersion of bagasse fibers within the polymeric matrix.
- DMA results included a complete analysis of the height and broadness of tan δ peaks and show that reprocessing and chemical modifications induced a better adhesion on the interface between bagasse fibers and PP matrix and increased the PP capacity to absorb energy.
- SEM micrographs show that during reprocessing the bagasse fibers got crushed to some extent and length was reduced around 20%. However it is important to remark that despite this decrease in fiber length, PP-Bag. biocomposites’ mechanical performance was maintained up to three reprocessing cycles without the addition of virgin material. Additionally, chemical treatments generated a bonding effect at the PP and natural fibers interface and exposed the cellulose of the bagasse, improving their dispersion within the polymeric matrix.
- Based on these findings, it could be concluded that bagasse fibers show an interesting potential for biocomposites production with a high potential of application in the design and manufacture of sustainable and highly recyclable products by injection molding. This could generate some economic and environmental benefits in the search for sustainability in the plastics industry.

## Figures and Tables

**Table 1:** Nomenclature of processed biocomposites.

Processing Cycle Number	Neat PP (wt.%)	Sugar Cane Bagasse (wt.%)	Chemical Treatment	Designation Along the Document
1	100	-	-	PP 1st cycle
	80	20	-	PP-Bag 1st cycle
			NaOH	PP-Bag +alk. 1st cycle
			NaOH + Silanes	PP-Bag +alk. +sil 1st cycle
3	100	-	-	PP 3rd cycle
	80	20	-	PP-Bag 3rd cycle
			NaOH	PP-Bag +alk. 3rd cycle
			NaOH + Silanes	PP-Bag +alk. +sil 3rd cycle
5	100	-	-	PP 5th cycle
	80	20	-	PP-Bag 5th cycle
			NaOH	PP-Bag +alk. 5th cycle
			NaOH + Silanes	PP-Bag +alk.+sil 5th cycle

**Table 2:** Flexural properties of PP and PP-Bagasse biocomposites.

Sample	Flexural Modulus (MPa)			Flexural Strength (MPa)		
	Processing Cycle Number					
	1	3	5	1	3	5
PP	1296 ± 70a	1251 ± 54a	1305 ± 36a	40.0 ± 0.7a	39.8 ± 0.9a	40.2 ± 0.4a
PP-Bag.	2069 ± 30b	1969 ± 48b	1673 ± 100b	48.0 ± 1.1b	44.1 ± 0.7b	41.3 ± 1.3a
PP-Bag.+alk.	1847 ± 114c	1853 ± 68c	1761 ± 78b	43.3 ± 0.5c	42.7 ± 0.3c	41.5 ± 1.2a
PP-Bag. +alk.+sil.	1505 ± 94d	1729 ± 66c	1742 ± 116b	38.6 ± 1.9a	41.2 ± 0.4d	40.4 ± 0.9a

(a–d) Different letters in the same column indicate significative differences from a set of means of each reprocessing group (*p* < 0.05).

**Table 3:** Thermal properties on cooling and second heating differential scanning calorimetry (DSC) scans of the samples.

Processing Cycle Number	Sample	Cooling	Second Heating		
		T_c_ * (°C)	T_m_ * (°C)	ΔH_m_ (J/g)	Χ_c_ (%)
1	PP	114	166	91	44
	PP-Bag.	122	163	75	45
	PP-Bag. +alk.	121	165	74	44
	PP-Bag. +alk. +sil.	118	166	99	59
3	PP	116	165	101	49
	PP-Bag.	119	165	80	49
	PP-Bag. +alk.	121	163	84	50
	PP-Bag. +alk. +sil.	122	165	100	60
5	PP	114	166	95	46
	PP-Bag.	121	164	75	46
	PP-Bag. +alk.	121	164	78	47
	PP-Bag. +alk. +sil.	121	162	83	50

* T_c_ and T_m_ were taken at the maximum peak of crystallization and melting peaks.

**Table 4:** Thermal degradation data of PP and PP-Bagasse biocomposites.

Processing Cycle	Sample	Degradation Stage	T_O_ (°C)	T_max_ (°C)
First cycle	PP	1	408	455
	PP-Bag.	1	271	354
		2	423	462
	PP-Bag. +alk.	1	314	363
		2	438	461
	PP-Bag. +alk. +sil.	1	317	360
		2	447	468
Third cycle	PP	1	373	445
	PP-Bag.	1	271	363
		2	431	463
	PP-Bag. +alk.	1	309	358
		2	431	468
	PP-Bag. +alk. +sil.	1	310	358
		2	428	457
Fifth cycle	PP	1	371	431
	PP-Bag.	1	287	366
		2	430	472
	PP-Bag. +alk.	1	318	360
		2	417	440
	PP-Bag. +alk. +sil.	1	322	360
		2	434	461

**Table 5:** Dynamic mechanical analysis (DMA) results of the studied materials.

Processing Cycle Number	Sample	Tg (°C) *	E′ (MPa) at 25 °C	Full Width at Half Maximum (FWHM) of Tan δ Peaks	Tan δ Peaks Height
1	PP	7.9	1481	24.4	0.091
	PP-Bag.	8.2	1741	23.3	0.083
	PP-Bag. +alk.	8.1	2261	21.9	0.081
	PP-Bag. +alk. +sil.	8.6	1962	22.2	0.081
3	PP	8.4	1453	24.9	0.087
	PP-Bag.	8.3	1810	22.5	0.087
	PP-Bag. +alk.	7.3	2319	20.7	0.078
	PP-Bag. +alk. +sil.	7.9	1835	20.5	0.080
5	PP	7.0	1383	23.4	0.093
	PP-Bag.	8.9	1905	22.7	0.083
	PP-Bag. +alk.	8.1	2111	21.1	0.080
	PP-Bag. +alk. +sil.	8.2	1899	21.8	0.078

* Tg values of the PP phase were taken at the maximum peak of the tan delta curves.
